# Effect of Different Restorative Materials and the Ferrule Height on the Biomechanical Behavior of Endocrowns in Endodontically Treated Maxillary and Mandibular Central Incisors: A Finite Element Analysis

**DOI:** 10.1155/ijod/4464923

**Published:** 2026-07-16

**Authors:** Sedigheh Sadat Hashemikamangar, Allahyar Geramy, Sara Valizadeh, Mohammadhossein Darayandeh, Behnaz Behniafar

**Affiliations:** ^1^ Department of Operative Dentistry, Tehran University of Medical Sciences, Tehran, Iran, tums.ac.ir; ^2^ Department of Orthodontics, Tehran University of Medical Sciences, Tehran, Iran, tums.ac.ir

**Keywords:** endocrown, ferrule, finite element analysis

## Abstract

**Background and Aims:**

Due to the advances in adhesive dentistry and the emphasis on less invasive principles, a treatment option for the restoration of endodontically treated teeth, called endocrown, has been presented. Using finite element as an in vitro simulation evaluation, this study investigated the effect of different restorative materials and the ferrule height on the biomechanical behavior of endocrowns in endodontically treated maxillary and mandibular central incisors.

**Materials and Methods:**

A sound maxillary central incisor and a mandibular central incisor were scanned and fed into Solidworks software for modeling. Endocrowns made of lithium disilicate, zirconia, zirconia‐reinforced lithium silicate, and Enamic were modeled by the program. After applying the forces, the maximum von Mises stress values were analyzed in the Ansys software.

**Results:**

The maximum von Mises stress on the endocrown, cement, enamel, and dentin of the maxillary central incisor was significantly higher than that on the endocrown, cementum, enamel, and dentin of the mandibular central incisor.

**Conclusion:**

It is recommended that a minimum ferrule be used in the central maxillary tooth to reduce the stress on the cementum. In the central mandibular tooth, using endocrowns made of Enamic is recommended because of less stress on the cementum.

## 1. Introduction

Restoring root canal‐treated teeth with extensive destruction has been challenging for most dentists over the years [1]. For decades, full‐coverage crowns with or without posts have been the golden choice of dentists for endodontically treated teeth [2]. However, due to radicular dentin loss, posts might weaken the remaining root structure and also cause iatrogenic root perforations. In addition, it has been shown that by harvesting the remaining healthy tissue during the preparation for full‐coverage restorations, the mechanical properties of the remaining tooth tissue are disrupted [3–5].

Anterior teeth are prone to deep diagonal fractures, especially in children. Residual roots and crowns after root treatment do not have a normal shape and have lost most of their structure. As a result, it is difficult to prepare a complete dentinal ferrule with sufficient clinical height for them. Therefore, restorations must function properly against severe stress [6]. To restore root canal‐treated anterior teeth, composite resins, ceramic veneers, metal‐ceramic crowns, and all‐ceramic crowns can be used according to the indications [7]. Some of the problems of direct restorations include fracture and microleakage caused by polymerization shrinkage [8].

With the advent of adhesive systems and dental materials that can be bonded to the tooth structure, such as ceramics, endocrowns are considered an alternative treatment option [6]. An endocrown is an integrated full‐crown restoration made of ceramic or reinforced composite resins that expands into the pulp chamber in multirooted teeth and the root canal in single‐rooted teeth and follows the principles of less invasive treatments. An endocrown obtains its macromechanical retention from the axial walls of the pulp chamber and its micromechanical and chemical retention from adhesive methods after being placed on the tooth [9, 10]. Currently, endocrown restorations are made using CAD/CAM technology, which allows one‐visit treatment. Compared to conventional, endocrowns offer a conservative restoration that prevents removing a large amount of sound tooth structure and requires a short manufacturing time.

In addition to these considerations, due to the essential role of the endocrown in the restoration of the coronal part, the endocrown properly maintains the seal of the radicular part, prevents bacterial microleakage, and positively affects the long‐term success of root canal‐treated teeth [11]. Furthermore, interventions are easier if the root canal treatment fails in teeth restored with an endocrown [12].

A direct method that can detect internal stress distribution is needed to better examine the distribution of stress in the restoration and tooth structure. The stresses inside a structure cannot be measured physically but can be calculated using the finite element analysis method [13, 14]. Currently, it is not clear whether an endocrown can provide favorable mechanical strength for the tooth‒restoration complex in cases with significant defects of the proximal wall and to what extent the dimensions of the proximal defects affect the fracture resistance of root canal‐treated teeth after endocrown restorations [15].

By considering all the aspects, it can be concluded that very little research has been performed on the central mandibular tooth. Also, no study has compared different endocrowns and the effect of ferrule thickness on the fracture resistance of endocrowns [1]. Studies in this field so far have mostly compared post‐and‐core crowns and endocrowns. In addition, studies on anterior teeth have mostly focused on maxillary central incisors [1, 16]. The objective of this study was to investigate the effect of various restorative materials and the extent of the ferrule on the biomechanical behavior of endocrowns fabricated for the endodontically treated maxillary and mandibular central incisors using finite element analysis.

## 2. Methods and Materials

### 2.1. Procedural Steps

This cross‐sectional observational study used finite element analysis and was performed without the involvement of any living tissue.

### 2.2. Scanning the Teeth and Preparing the Models

One sound maxillary central incisor and one mandibular central incisor, extracted due to periodontitis, were obtained from the Department of Surgery. Using the ATOS CORE scanner (GOM Co., Germany), these two teeth were accurately scanned, and the scanned file was imported to SolidWorks 2021 (Dassault Systemes S. France) modeling software. For ideal simulation, the alveolar bone was also designed. To simulate the oral tissues, a 0.25 mm periodontal membrane and the surrounding cortical bone and cancellous bone with a 2 mm thickness were simulated. First, the root canals of both teeth were obturated with gutta percha.

**Figure 1:**
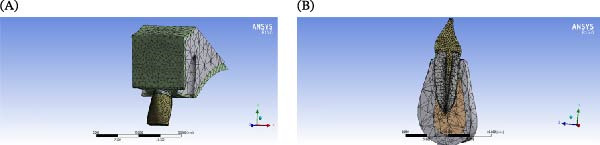
Upper central incisor meshed model and lower central incisor meshed model. (A) Upper central incisor meshed model with 1 mm height of ferrule. (B) Lower central incisor meshed model with 0.5 mm height of ferrule.

The modeling was performed as follows:

For the maxillary central incisor, reduction was carried out to create three models with ferrule heights of 1, 2, and 3 mm (Figure 1A).
For the mandibular central incisor, reduction was performed to create three models with ferrule heights of 0.5, 1, and 1.5 mm (Figure 1B).
Endocrowns were modeled using the following materials:
Lithium disilicate (IPS e.max).
Enamic.
Zirconia‐reinforced lithium silicate.

### 2.3. Assumptions and Test Conditions

1. Endocrowns were cemented to the teeth with Panavia cement with a thickness of 0.1 mm.
2. The penetration depth of endocrowns into the root canal was set at 4 mm.
3. The thickness of the ferrule in the maxillary central incisor was 1 mm and 0.5 in the mandibular central incisor.
4. 1 mm of enamel wall above the CEJ was considered for all the models.

### 2.4. Finite Element Analysis in the ANSYS Software

The following conditions were considered for the model.

1. The materials used in all the models were considered homogeneous, isotropic, and linearly elastic.
2. The default in finite element analysis is that the models are placed without any defect in the alveolar bone and do not move in any direction.
3. The components were considered without defects.

After preparing the models, numerical analysis was performed using the static structural method using the ANSYS software.

### 2.5. Allocation of Material

In this software, after importing the models from the SolidWorks software, the material of each part must be determined based on the modulus of elasticity and Poisson’s coefficient. The mechanical information of the materials was extracted from Table 1.

**Table 1:** Poisson’s ratios and Young’s moduli of studied dental materials and tooth structures.

Materials and tooth structures	Young’s elastic modulus/GPa	Poisson’s ratio (v)
Dentin	18.6	0.31
Enamel	84.1	0.33
Cementum	2.4	0.31
Periodontal ligament	0.069	0.45
Cortical bone	13.7	0.3
Trabecular bone	1.37	0.3
Gutta‐percha	0.00069	0.45
Panavia cement	18.6	0.28
IPS e. max lithium disilicate	95	0.3
Vita Enamic	37.8	0.24
Zirconia reinforced lithium silicate	70	0.23

### 2.6. Contact Definition

In this step, bonded contact was defined between the components of the sets. In fact, in one model, both parts placed on top of each other or next to each other are fix‐bonded. For example, the enamel is inseparably bonded to the underlying dentin.

### 2.7. Meshing

Then, the meshing of the models was performed. Table 2 presents the number of elements and nodes of the models.

**Table 2:** The total number of elements and nodes of the models.

Models	Node number	Element number
0.5 mm, Lower central incisor	54,111	30,155
1 mm, Lower central incisor	51,329	27,804
1.5 mm, Lower central incisor	60,530	33,054
1 mm, Upper central incisor	87,400	50,098
2 mm, Upper central incisor	92,011	51,402
3 mm, Upper central incisor	101,250	55,682

### 2.8. Boundary Condition Definition of the Models

In the next step, for the boundary conditions of the upper surface of the central maxillary models and the lower surface of the central mandibular models, they were prevented from moving in all directions in the cortical and spongy bones.

### 2.9. Applying Force

The maximum masticatory force in the maxillary and mandibular central incisors was 120 Newtons, which was applied to the teeth [17]. The force was applied to the maxillary central incisor at a 25° angle to the long axis of the tooth between the middle and incisal thirds of the lingual surface with a 2 mm^2^ surface area. A force was applied to the mandibular central incisor along the long axis of the tooth at the incisal edge with a cross‐section of 1 mm^2^.

## 3. Results

Tables 3–6 and Figures 2–5 present the maximum von Mises stresses in endocrowns, cementum, enamel, and dentin.

Figure 2Maximum von Mises stress in endocrown. (A) Upper central incisor with 1 mm height of ferrule restored with IPS e. max lithium disilicate endocrown. (B) Upper central incisor with 2 mm height of ferrule restored with IPS e. max lithium disilicate endocrown. (C) Upper central incisor with 3 mm height of ferrule restored with IPS e. max lithium disilicate endocrown. (D) Upper central incisor with 1 mm height of ferrule restored with zirconia endocrown. (E) Upper central incisor with 2 mm height of ferrule restored with zirconia endocrown. (F) Upper central incisor with 3 mm height of ferrule restored with zirconia endocrown. (G) Upper central incisor with 1 mm height of ferrule restored with Vita Enamic endocrown. (H) Upper central incisor with 2 mm height of ferrule restored with Vita Enamic endocrown. (I) Upper central incisor with 3 mm height of ferrule restored with Vita Enamic endocrown. (J) Upper central incisor with 1 mm height of ferrule restored with zirconia‐reinforced lithium silicate. (K) Upper central incisor with 2 mm height of ferrule restored with zirconia‐reinforced lithium silicate. (L) Upper central incisor with 3 mm height of ferrule restored with zirconia‐reinforced lithium silicate. (M) Lower central incisor with 0.5 mm height of ferrule restored with IPS e. max lithium disilicate endocrown. (N) Lower central incisor with 1 mm height of ferrule restored with IPS e. max lithium disilicate endocrown. (O) Lower central incisor with 1.5 mm height of ferrule restored with IPS e. max lithium disilicate endocrown. (P) Lower central incisor with 0.5 mm height of ferrule restored with zirconia endocrown. (Q) Lower central incisor with 1 mm height of ferrule restored with zirconia endocrown. (R) Lower central incisor with 1.5 mm height of ferrule restored with zirconia endocrown. (S) Lower central incisor with 0.5 mm height of ferrule restored with Vita Enamic endocrown. (T) Lower central incisor with 1 mm height of ferrule restored with Vita Enamic endocrown. (U) Lower central incisor with 1.5 mm height of ferrule restored with Vita Enamic endocrown. (V) Lower central incisor with 0.5 mm height of ferrule restored with zirconia‐reinforced lithium silicate endocrown. (W) Lower central incisor with 1 mm height of ferrule restored with zirconia‐reinforced lithium silicate endocrown. (X) Lower central incisor with 1.5 mm height of ferrule restored with zirconia‐reinforced lithium silicate endocrown.

**Figure:**
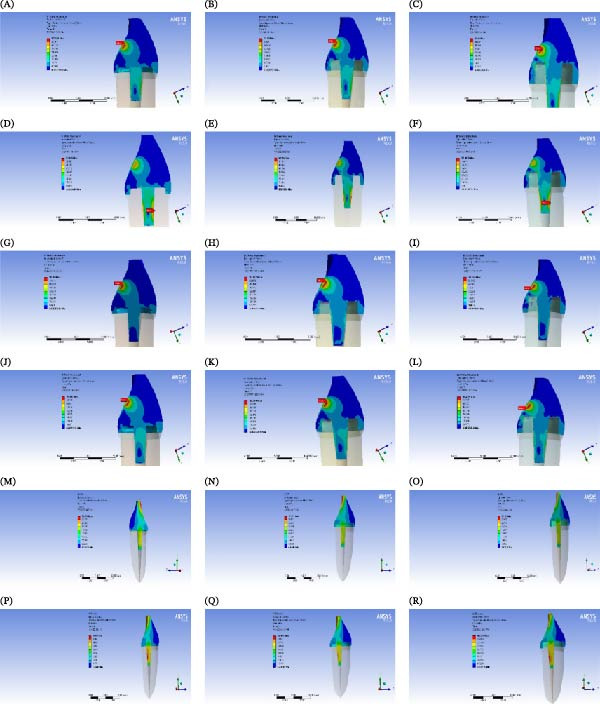

**Figure:**
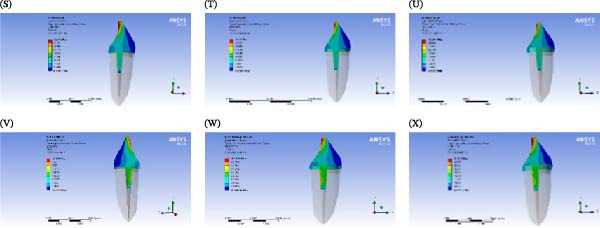

Figure 3Maximum von Mises stress in cementum. (A) Upper central incisor with 1 mm height of ferrule restored with IPS e. max lithium disilicate endocrown. (B) Upper central incisor with 2 mm height of ferrule restored with IPS e. max lithium disilicate endocrown. (C) Upper central incisor with 3 mm height of ferrule restored with IPS e. max lithium disilicate endocrown. (D) Upper central incisor with 1 mm height of ferrule restored with zirconia endocrown. (E) Upper central incisor with 2 mm height of ferrule restored with zirconia endocrown. (F) Upper central incisor with 3 mm height of ferrule restored with zirconia endocrown. (G) Upper central incisor with 1 mm height of ferrule restored with Vita Enamic endocrown. (H) Upper central incisor with 2 mm height of ferrule restored with Vita Enamic endocrown. (I) Upper central incisor with 3 mm height of ferrule restored with Vita Enamic endocrown. (J) Upper central incisor with 1 mm height of ferrule restored with zirconia‐reinforced lithium silicate. (K) Upper central incisor with 2 mm height of ferrule restored with zirconia‐reinforced lithium silicate. (L) Upper central incisor with 3 mm height of ferrule restored with zirconia‐reinforced lithium silicate. (M) Lower central incisor with 0.5 mm height of ferrule restored with IPS e. max lithium disilicate endocrown. (N) Lower central incisor with 1 mm height of ferrule restored with IPS e. max lithium disilicate endocrown. (O) Lower central incisor with 1.5 mm height of ferrule restored with IPS e. max lithium disilicate endocrown. (P) Lower central incisor with 0.5 mm height of ferrule restored with zirconia endocrown. (Q) Lower central incisor with 1 mm height of ferrule restored with zirconia endocrown. (R) Lower central incisor with 1.5 mm height of ferrule restored with zirconia endocrown. (S) Lower central incisor with 0.5 mm height of ferrule restored with Vita Enamic endocrown. (T) Lower central incisor with 1 mm height of ferrule restored with Vita Enamic endocrown. (U) Lower central incisor with 1.5 mm height of ferrule restored with Vita Enamic endocrown. (V) Lower central incisor with 0.5 mm height of ferrule restored with zirconia‐reinforced lithium silicate endocrown. (W) Lower central incisor with 1 mm height of ferrule restored with zirconia‐reinforced lithium silicate endocrown. (X) Lower central incisor with 1.5 mm height of ferrule restored with zirconia‐reinforced lithium silicate endocrown.

**Figure:**
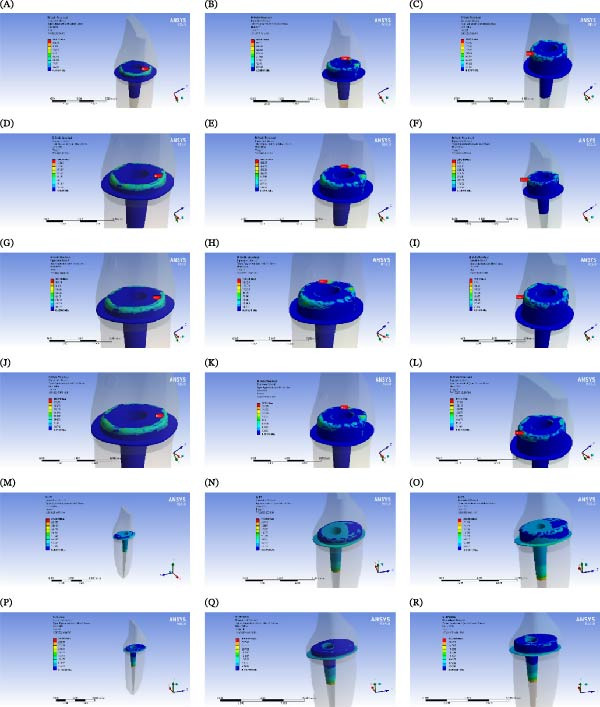

**Figure:**
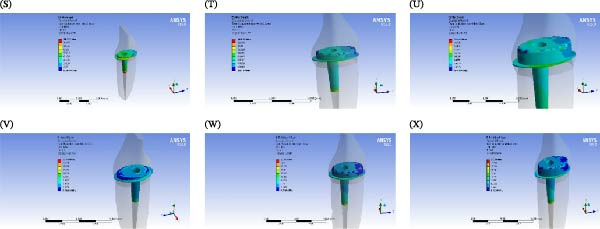

Figure 4Maximum von Mises stress in enamel. (A) Upper central incisor with 1 mm height of ferrule restored with IPS e. max lithium disilicate endocrown. (B) Upper central incisor with 2 mm height of ferrule restored with IPS e. max lithium disilicate endocrown. (C) Upper central incisor with 3 mm height of ferrule restored with IPS e. max lithium disilicate endocrown. (D) Upper central incisor with 1 mm height of ferrule restored with zirconia endocrown. (E) Upper central incisor with 2 mm height of ferrule restored with zirconia endocrown. (F) Upper central incisor with 3 mm height of ferrule restored with zirconia endocrown. (G) Upper central incisor with 1 mm height of ferrule restored with Vita Enamic endocrown. (H) Upper central incisor with 2 mm height of ferrule restored with Vita Enamic endocrown. (I) Upper central incisor with 3 mm height of ferrule restored with Vita Enamic endocrown. (J) Upper central incisor with 1 mm height of ferrule restored with zirconia‐reinforced lithium silicate. (K) Upper central incisor with 2 mm height of ferrule restored with zirconia‐reinforced lithium silicate. (L) Upper central incisor with 3 mm height of ferrule restored with zirconia‐reinforced lithium silicate. (M) Lower central incisor with 0.5 mm height of ferrule restored with IPS e. max lithium disilicate endocrown. (N) Lower central incisor with 1 mm height of ferrule restored with IPS e. max lithium disilicate endocrown. (O) Lower central incisor with 1.5 mm height of ferrule restored with IPS e. max lithium disilicate endocrown. (P) Lower central incisor with 0.5 mm height of ferrule restored with zirconia endocrown. (Q) Lower central incisor with 1 mm height of ferrule restored with zirconia endocrown. (R) Lower central incisor with 1.5 mm height of ferrule restored with zirconia endocrown. (S) Lower central incisor with 0.5 mm height of ferrule restored with Vita Enamic endocrown. (T) Lower central incisor with 1 mm height of ferrule restored with Vita Enamic endocrown. (U) Lower central incisor with 1.5 mm height of ferrule restored with Vita Enamic endocrown. (V) Lower central incisor with 0.5 mm height of ferrule restored with zirconia‐reinforced lithium silicate endocrown. (W) Lower central incisor with 1 mm height of ferrule restored with zirconia‐reinforced lithium silicate endocrown. (X) Lower central incisor with 1.5 mm height of ferrule restored with zirconia‐reinforced lithium silicate endocrown.

**Figure:**
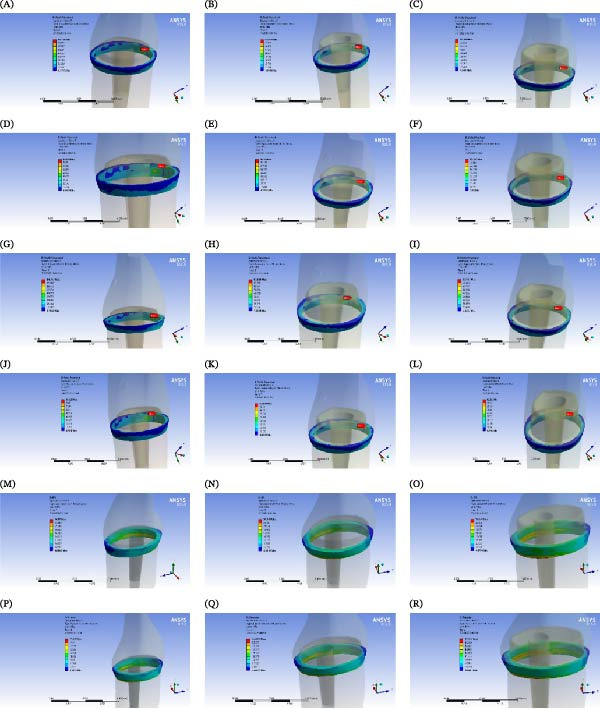

**Figure:**
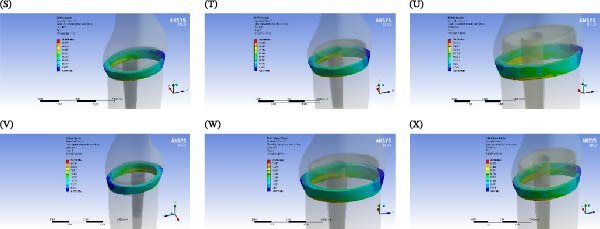

Figure 5Maximum von Mises stress in dentin. (A) Upper central incisor with 1 mm height of ferrule restored with IPS e. max lithium disilicate endocrown. (B) Upper central incisor with 2 mm height of ferrule restored with IPS e. max lithium disilicate endocrown. (C) Upper central incisor with 3 mm height of ferrule restored with IPS e. max lithium disilicate endocrown. (D) Upper central incisor with 1 mm height of ferrule restored with zirconia endocrown. (E) Upper central incisor with 2 mm height of ferrule restored with zirconia endocrown. (F) Upper central incisor with 3 mm height of ferrule restored with zirconia endocrown. (G) Upper central incisor with 1 mm height of ferrule restored with Vita Enamic endocrown. (H) Upper central incisor with 2 mm height of ferrule restored with Vita Enamic endocrown. (I) Upper central incisor with 3 mm height of ferrule restored with Vita Enamic endocrown. (J) Upper central incisor with 1 mm height of ferrule restored with zirconia‐reinforced lithium silicate. (K) Upper central incisor with 2 mm height of ferrule restored with zirconia‐reinforced lithium silicate. (L) Upper central incisor with 3 mm height of ferrule restored with zirconia‐reinforced lithium silicate. (M) Lower central incisor with 0.5 mm height of ferrule restored with IPS e. max lithium disilicate endocrown. (N) Lower central incisor with 1 mm height of ferrule restored with IPS e. max lithium disilicate endocrown. (O) Lower central incisor with 1.5 mm height of ferrule restored with IPS e. max lithium disilicate endocrown. (P) Lower central incisor with 0.5 mm height of ferrule restored with zirconia endocrown. (Q) Lower central incisor with 1 mm height of ferrule restored with zirconia endocrown. (R) Lower central incisor with 1.5 mm height of ferrule restored with zirconia endocrown. (S) Lower central incisor with 0.5 mm height of ferrule restored with Vita Enamic endocrown. (T) Lower central incisor with 1 mm height of ferrule restored with Vita Enamic endocrown. (U) Lower central incisor with 1.5 mm height of ferrule restored with Vita Enamic endocrown. (V) Lower central incisor with 0.5 mm height of ferrule restored with zirconia‐reinforced lithium silicate endocrown. (W) Lower central incisor with 1 mm height of ferrule restored with zirconia‐reinforced lithium silicate endocrown. (X) Lower central incisor with 1.5 mm height of ferrule restored with zirconia‐reinforced lithium silicate endocrown.

**Figure:**
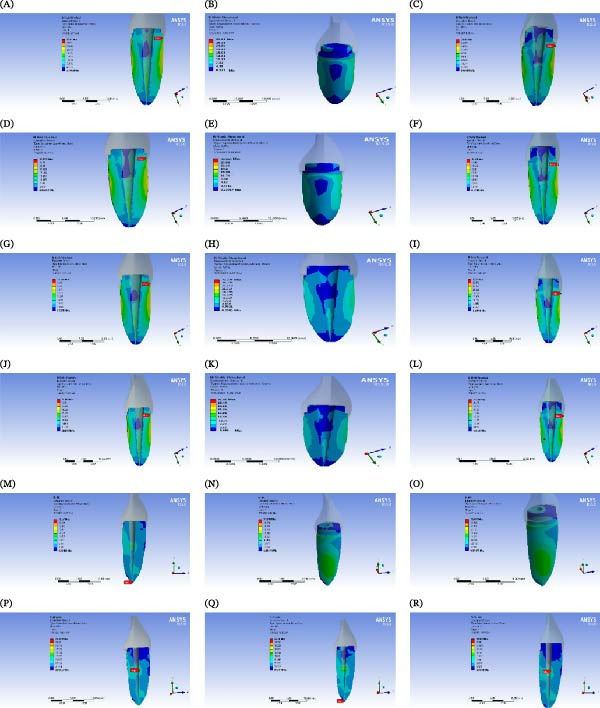

**Figure:**
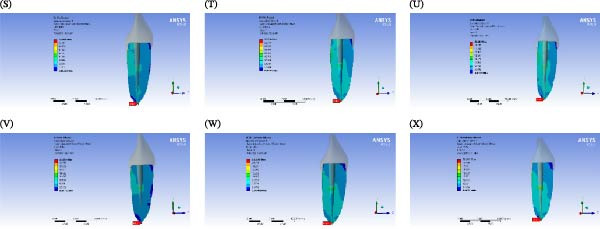

**Table 3:** Maximum von Mises stresses in endocrowns in MPa.

		IPS e. max lithium disilicate	Vita Enamic	Zirconia‐reinforced lithium silicate
Upper incisor	1 mm height of ferrule	57.281	59.09	59.386
	2 mm height of ferrule	57.251	59.121	59.394
	3 mm height of ferrule	56.46	58.176	58.237
Lower incisor	0.5 mm height of ferrule	33.174	33.276	33.301
	1 mm height of ferrule	33.227	33.32	33.347
	1.5 mm height of ferrule	33.119	33.211	33.239

**Table 4:** Maximum von Mises stress in cementum in MPa.

		IPS e. max lithium disilicate	Vita Enamic	Zirconia‐reinforced lithium silicate
Upper incisor	1 mm height of ferrule	146.73	147.48	147.01
	2 mm height of ferrule	183.01	176.98	181.8
	3 mm height of ferrule	199.21	192.93	195.5
Lower incisor	0.5 mm height of ferrule	24.862	18.722	22.753
	1 mm height of ferrule	25.496	18.816	23.165
	1.5 mm height of ferrule	24.453	18.434	22.396

**Table 5:** Maximum von Mises stress in enamel in MPa.

		IPS e. max lithium disilicate	Vita Enamic	Zirconia‐reinforced lithium silicate
Upper incisor	1 mm height of ferrule	85.159	84.767	85.022
	2 mm height of ferrule	83.619	83.665	83.609
	3 mm height of ferrule	82.484	82.032	82.323
Lower incisor	0.5 mm height of ferrule	26.05	30.869	27.768
	1 mm height of ferrule	26.745	30.948	28.295
	1.5 mm height of ferrule	24.647	28.78	26.168

**Table 6:** Maximum von Mises stress in dentin in MPa.

		IPS e. max lithium disilicate	Vita Enamic	Zirconia‐reinforced lithium silicate
Upper incisor	1 mm height of ferrule	32.01	32.503	32.182
	2 mm height of ferrule	31.61	32.236	31.91
	3 mm height of ferrule	31.45	32.03	31.653
Lower incisor	0.5 mm height of ferrule	25.07	25.068	25.069
	1 mm height of ferrule	23.258	23.256	23.258
	1.5 mm height of ferrule	23.815	22.101	22.103

The summary of the results of the present study using the finite element analysis is as follows.

1. The maximum von Mises stress on the endocrown, cementum, enamel, and dentin in the maxillary central incisor was greater than that in the central incisor of the mandible.
2. The maximum von Mises stress on the maxillary central incisor endocrowns made of Enamic and zirconia‐reinforced lithium silicate was higher than endocrowns made of lithium disilicate.
3. The maximum von Mises stress on the mandibular central incisor endocrowns made of Enamic, zirconia‐reinforced lithium silicate, and lithium disilicate were not significantly different.
4. The amount of ferrule did not affect the maximum von Mises stress in the maxillary and mandibular central incisor endocrowns in any of the manufactured endocrowns.
5. The endocrown material did not affect the maximum von Mises stress on the cementum in the maxillary central incisor tooth.
6. The amount of ferrule affected the maximum von Mises stress on the cementum in the maxillary central incisor tooth. The maximum Von Mises stress was observed with the maximum ferrule, and the minimum von Mises stress was observed with the minimum ferrule.
7. Endocrowns material affected the maximum von Mies stress on the cementum in the mandibular central incisor tooth, and the maximum stress on the cementum was observed, in descending order, in endocrowns made of lithium disilicate, zirconia‐reinforced lithium silicate, and Enamic.
8. The amount of ferrule did not affect the maximum von Mises stress on the cementum in the mandibular central incisor tooth.
9. The endocrown material and the amount of ferrule did not affect the maximum von Mises stress on the enamel in the maxillary central incisor tooth.
10. The endocrown material affected the maximum von Mises stress on the enamel in the mandibular central incisor tooth, and the maximum von Mises stress on the enamel was observed in endocrowns made of Enamic, with no significant difference between the other endocrowns.
11. The amount of ferrule did not affect the maximum von Mises stress on the enamel in the mandibular central incisor tooth.
12. The amount of ferrule and endocrown material did not affect the maximum von Mises stress on dentin in the maxillary and mandibular central incisor teeth.

## 4. Discussion

Many factors can affect the maximum von Mises stress in the maxillary and mandibular central incisor teeth with endocrowns. Studies show that some factors can affect von Mises stress, such as ferrule height, amount of force, angle of force applied to the tooth, location of force application, and the endocrown materials [17]. Results of this study indicated that some major factors have effect on von Mises stress of endocrowns on maxillary and mandibular teeth.

### 4.1. Influence of Tooth Size and Anatomy:Anatomy

As stated in the results of this study, the finite element stress of all structures in the maxillary central incisor was greater than in the central incisor of the mandible which could be explained by the size and direction of surface area that has been under force [18]. The maximum stress in endocrowns made of Enamic and zirconia‐reinforced lithium silicate was higher than lithium disilicate in the maxillary central incisor, and the maximum stress in all three materials did not show any significant changes in the mandibular incisor, which could be explained by the direction, size and position area, and maximum force applied to each tooth similar to the finding of Soliman et al. [19]. Although studies such as Naumann et al. [20] stated that endocrown materials with modulus of elasticity near to tooth structure, such as Enamics, show significantly better stress distribution on applied forces.

### 4.2. Influence of Ferrule Height

The amount of ferrule only affected the cementum of the maxillary central incisor, which could be explained by the amount of cementum tissue thickness and the luting cement thickness in the cervical area of the maxillary tooth and the direction and amount of force applied [21]. As stated by Bindl and Mormann, endocrowns are able to distribute occlusal forces more evenly [22], but sufficient bonding space has a significant effect in the retention and stabilization of endocrowns due to the smaller pulp chamber area in the incisors, which may reduce the retention forces [23].

### 4.3. Influence of Different Endocrown Materials

Endocrowns materials only affected the maximum Von Mies stress on the cementum and enamel in the mandibular central incisor tooth, which could be explained by the findings of Zarone et al. [24], which indicates that the location of force values of stresses on each model component changed the load location, perhaps on the root, cement, and endocrown due to changing the load transfer mechanism, as ratios between bending and shear stresses changed. These results also indicate that highly elastic modulus ceramics—zirconium or aluminum oxide should not be used in endocrowns fabrication due to their insufficient bonding to the dentin, which might induce concentrated high‐stress at the endocrown–cement–dentin connections and in conclusion, tooth remnants should be saved and are quite important [25].

As previously stated, a good and complete dentin ferrule is necessary for a post‐and‐crown or fiber post‐and‐crown restoration of an endodontically treated maxillary central incisor with a severe structural defect. When this dentin ferrule is not available in sufficient quantity, stress distribution in the endocrown is better than in the post‐and‐core crown [6] although some studies indicate that at least 1–2 mm of ferrule in endocrowns may increase the bonding surface for good adhesion of endocrowns [26].

Li et al. applied finite element analysis to compare the stress distribution in endodontically treated maxillary central incisors treated with endocrown, fiber post‐and‐core crown, or cast post‐and‐core crown restorations. The results showed that endocrown was the most suitable restoration for these teeth [6], in contrast to a study by Dejak and Mlotkowski [16] finite element analysis, which showed that post‐and‐core crown was the most suitable restoration compared to endocrown. In line with the findings of the present study, Heintze et al. [27] reported that hybrid ceramic materials like Enamics, preserve better stress distribution and lower von Mises stress than zirconia‐reinforced ceramics. Besides these results, Kelly and Benetti [28] reported that aesthetic and biomechanics should be considered in anterior teeth with high stress concentration. Crown materials have a crucial effect in biomechanical performance. As Soliman et al. [19] reported, Vita Enamic had lower stress concentrations compared to Celtra Duo at the finish line of crowns, suggesting that they would be the better option for reducing the risk of fractures and enhancing the durability of restorations. However, Celtra Duo also had better findings, particularly in endocrowns, indicating that they can be a viable option in specific clinical requirements [19]. Waaz compared endocrowns with different depths made of zirconia‐reinforced lithium silicate with post‐and‐core crowns made of zirconia‐reinforced lithium silicate. The results showed that the endocrown with low extension into the root canal was the best restoration [1]. Maximum von Mises stress in maxillary central incisor endocrowns was recorded at the site of force application on the lingual surface near the cingulum, which did not coincide with the findings reported by Li et al. [6] and Dejak and Mlotkowski [16]. The maximum Von Mises stress was observed in the mandibular central incisor endocrowns at the force application site at the incisal edge. The maximum von Mises stress applied to the cementum in the central maxillary incisor endocrowns was observed at the site of connection of the ferrule tip to the cementum; however, in Li et al. [6] study, it was observed at the tip of the endocrown retainer. The maximum von Mises stress applied to the cementum in the mandibular central incisor endocrowns was observed at the tip of the endocrown retainer.

The endocrown material had no effect on the maximum von Mises stress on the cementum in the maxillary central incisor tooth. However, the amount of ferrule significantly affected the maximum von Mises stress on the cementum in the maxillary central incisor tooth. The maximum von Mises stress was observed with the maximum ferrule, and the minimum von Mises stress was observed with the minimum ferrule. Therefore, a minimal ferrule is recommended to reduce the stress on the cementum. Although finite element analysis is a feasible mechanical analysis but it cannot fully simulate the oral cavity condition and masticatory dynamic forces. Variable factors in oral cavity are periodontal considerations, bone density alterations, and different masticatory forces in each specific patient that indicates the urgent need of further clinical and advanced in vitro studies. As mentioned previously, no study has compared different endocrowns and the effect of ferrule thickness on the fracture resistance of endocrowns to compare with our study in discussion. The findings of our study were valuable for maxillary and mandibular incisor teeth, but considerations should be taken for posterior teeth such as premolars and molars in both jaws to enhance the applicability of the results across different clinical scenarios. Additionally, further research should concentrate on other real factors such as morphology of each tooth, occlusal loading patterns, real oral cavity situations, and the presence of adjacent teeth on stress distribution. By considering these situations, these finite element studies can provide more reliable findings that influence the success and longevity of restorations in endodontically treated teeth.

## 5. Conclusion

The endocrown material had a significant effect on the maximum von Mises stress on the cementum in the mandibular central incisor tooth, and the maximum stress on the cementum was recorded in descending order as follows: endocrowns made of lithium disilicate, zirconia‐reinforced lithium silicate, and Enamic. Therefore, endocrowns with lower maximum stress on cementum, like Enamic, are recommended.

## Author Contributions

**Sedigheh Sadat Hashemikamangar, Allahyar Geramy, Sara Valizadeh, and Mohammadhossein Darayandeh:** conceptualization, methodology, writing – original draft preparation, software, project administration, interpretation of the data. **Behnaz Behniafar:** responsible for manuscript submission and responding to reviewers’ comments, writing – final draft preparation and supervision. All authors: writing – review and editing.

## Funding

This study did not receive any specific funding.

## Disclosure

The authors state that this manuscript has been previously published in preprint format [29]. This study was conducted on extracted teeth due to periodontal problems. This finite element analysis was performed without the involvement of any living tissue.

## Ethics Statement

The ethical aspects of this study were reviewed and approved by the Ethics Committee of the Faculty of Dentistry, Tehran University of Medical Sciences (with ethical code of IR.TUMS.DENTISTRY.REC.1401.001).

## Consent

The authors have nothing to report.

## Conflicts of Interest

The authors declare no conflicts of interest.

## Data Availability

All data support the findings of this study are available within the content of the manuscript.
